# Comparison of Mechanical Responses of Asphalt Mixtures under Uniform and Non-Uniform Loads Using Microscale Finite Element Simulation

**DOI:** 10.3390/ma12193058

**Published:** 2019-09-20

**Authors:** Guoyang Lu, Chonghui Wang, Pengfei Liu, Stefan Pyrek, Markus Oeser, Sabine Leischner

**Affiliations:** 1 Institute of Highway Engineering, RWTH Aachen University, D52074 Aachen, Germany; lu@isac.rwth-aachen.de (G.L.); c.wang@isac.rwth-aachen.de (C.W.); oeser@isac.rwth-aachen.de (M.O.); 2 Institute for Automotive Engineering, RWTH Aachen University, D52074 Aachen, Germany; stefan.pyrek@ika.rwth-aachen.de; 3 Institute for Urban and Pavement Engineering, Technical University of Dresden, D01187 Dresden, Germany

**Keywords:** asphalt mixture, non-uniform load, microstructure, digital image processing, finite element method

## Abstract

Continuously increasing traffic volumes necessitate accurate design methods to ensure the optimal service life and efficient use of raw materials. Numerical simulations commonly pursue a simplified approach with homogeneous pavement materials and homogeneous loading. Neither the pavement geometry nor the loading is homogeneous in reality. In this study, the mechanical response of the asphalt mixtures due to homogeneous loads is compared with their mechanical response to inhomogeneous loads. A 3D finite element model was reconstructed with the aid of X-ray computed tomography. Sections of a real tire’s pressure distribution were used for the inhomogeneous loads. The evaluation of the material response analyzes the stress distribution within the samples. An inhomogeneous load evokes an increased proportion of high stresses within the sample in every case, particularly at low temperatures. When comparing the two types of loads, the average stresses on the interior (tension and compression) exhibit significant differences. The magnitude of the discrepancies shows that this approach yields results that differ significantly from the common practice of using homogeneous models and can be used to improve pavement design.

## 1. Introduction

The steadily increased traffic load reinforces the need for precise assessment of the pavement to ensure optimal use of increasingly scarce resources. Numerical simulation methods, such as finite element method (FEM) and discrete element method (DEM), represent the currently chosen approaches [1,2,3,4]. One of the most important factors in FEM modeling of flexible pavements is the geometry of contact area between tire and pavement as well as the loading distribution. The pressure of a tire is transferred to the pavement layers through the contact area. The structural response of a flexible pavement is sensitive to the contact area and loading distribution, especially in hot climatic areas [5]. Different contact areas and loading distribution may lead to different influences on distress of the pavement near-surface. Both of them depend essentially on tire type, tire load, and tire inflation pressure [6].

Traffic load is composed of three components in vertical, transversal, and longitudinal directions, respectively. The vertical component of the load is significantly greater than the transversal and longitudinal ones. The horizontal components of load (transversal and longitudinal) may only be significant during braking or turning operations. As a result, normally pavement designers only regard the vertical component, which requires less experimental measurements and computational effort [7].

There are several methods to estimate the magnitude of the vertical load component. The simpler procedure is to divide the vertical load by the tire–pavement contact area, and thus an average and uniform vertical stress is obtained. However, De Beer and Jooste [8] indicated that this procedure is not suitable for thin pavements with thickness less than 100 mm. They proposed an expression that depends on tire inflation pressure to estimate the value of the vertical contact stress. Other researchers such as Steyn and Visser [9] also proposed expressions for this purpose, which depended not only on tire inflation pressure, but also on the tire type and the tire load. These methods estimated the vertical contact stress disregarding the non-uniform stress distribution. However, Tielking and Roberts [10] pointed out that the non-uniform distribution of the real pavement contact stresses existed during vehicle movement due to tire deformation. An early research by Jooste and Lourens [11] and a detailed study by Groenendijk [12] indicated that the non-uniformity of the contact stresses significantly influenced the development and magnitude of the tensile strain in flexible pavement systems. Their studies also showed that the conventional multilayer elastic (MLE) models, with a uniform circular contact stress potentially underestimate the maximum horizontal tensile strain.

Blab assessed three-dimensional (3D) contact stresses occurring between the road surface and three tire types (pneumatic bias ply, radial, and wide base) by means of the South African Vehicle Road Surface Pressure Transducer Array (VRSPTA) device in spring 1997 [13]. Measurements were performed under different load and tire inflation pressure conditions at relatively low speed ranges. The results of a comprehensive statistical study on tire geometry and measured contact stresses of the tire imprints were described. Based on this study, regression functions were introduced to estimate both forces and average contact stresses occurring at the tire center and tire edge. Proposals were presented to utilize these regression functions for improved tire load models for pavement analysis. Based on the previous experimental investigations, early studies on FEM modeling of flexible pavements were carried out [14,15,16]. For example, Blab and Harvey conducted FEM simulation of pavement performance under the Heavy Vehicle Simulator (HVS) using a linear viscoelastic model, with the nodal forces in the longitudinal, transverse, and vertical directions measured by VRSPTA to provide more realistic loading rather than an assumed circular uniform stress [14]. These FEM studies indicated that the regular shape of the contact area such as a rectangle with non-uniform loading distribution indeed improved prediction of the stress and strain state in the near-surface pavement section. Development of significant tensile strains and stresses was found to be close to the outside of the tire edge, and compressive stresses and strains inside the tire contact patch; highly non-uniformly distributed stress and strain patterns were identified in the top 50–75 mm of the pavement.

With the development of measuring methods and computer technology, the non-uniformly distributed pressure and irregular contact areas have been applied in FEM modeling of the pavement. Novak et al. [17] used the FEM code ADINA to model the 3D effects of measured tire contact stresses in a typical pavement configuration. The results showed that the predicted stress states and measured radial tire contact stresses were both larger in magnitude and more focused near the surface than those obtained from traditional circular uniform vertical loading conditions. In terms of effects of possible pavement damage mechanisms, predicted high near-surface shear stresses may be a part of an explanation for near-surface rutting failure modes. Wang et al. [18] constructed a 3D tire-pavement interaction model in ABAQUS (Version 6.7, Dassault Systèmes Simulia Corp., Providence, RI, United States) to predict the contact stress distributions for future use in the mechanistic analysis of pavement responses. The ribbed radial-ply tire was modeled as a composite structure (rubber and reinforcement), and the tire material parameters were calibrated through load-deflection curves. The steady-state tire rolling process was simulated using an arbitrary Lagrangian–Eulerian (ALE) formulation. The model results were consistent with previous measurements and validated the existence of non-uniform vertical contact stresses and localized tangential contact stresses. The analysis results showed that the non-uniformity of vertical contact stresses decreased as the load increased, but increased as the inflation pressure increased. Vehicle maneuvering behavior significantly affected the tire–pavement contact stress distributions. The model results provided valuable insights into understanding the realistic tire–pavement interaction for analyzing pavement responses at critical loading conditions.

In order to derive more accurate prediction from the FEM simulation, the non-uniform load distribution should be considered in further FEM modeling of asphalt pavement materials according to the aforementioned literature study. At the mesoscale and microscale, asphalt mixtures are typically heterogeneous materials consisting of aggregate, asphalt binder, and air voids. During the last two decades, the microstructure of asphalt mixtures has been reconstructed in different ways to allow for more realistic numerical simulations [19]. The results derived from heterogeneous models are closer to experimental results and represent the reality more precisely [20]. In this study, the digital image processing (DIP) technique was used to reconstruct heterogeneous FEM models of asphalt mixtures. The uniform and non-uniform loads were applied on the heterogeneous FEM models. The mechanical responses of the asphalt mixtures were compared and evaluated.

## 2. Research Methodology

### 2.1. Preparation of Asphalt Samples

**Figure 1:**
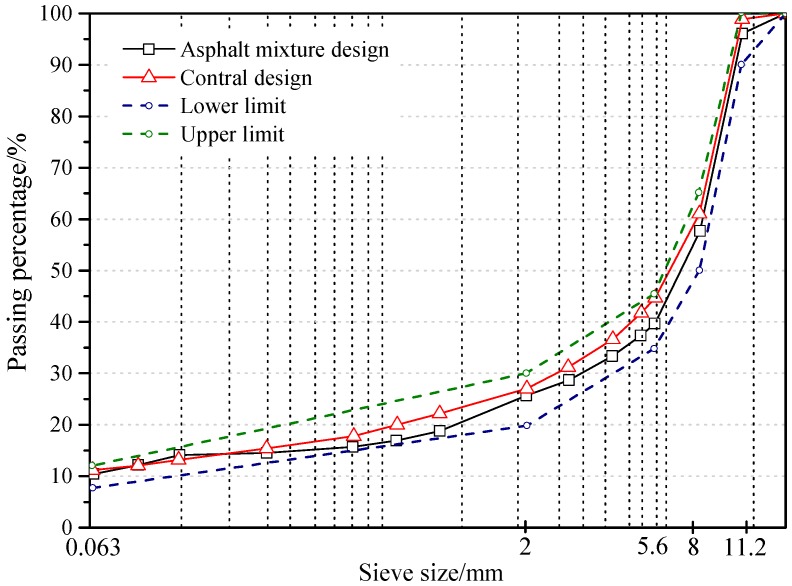
Grading curve of stone mastic asphalt with maximum grain size of 11 mm (SMA-11S).

For the samples a stone mastic asphalt (SMA) with a maximum grain size of 11 mm (SMA-11S) was prepared. The aggregate consisted of diabase with a grain size distribution as shown in Figure 1.

The selected binder was unmodified penetration graded bitumen of the type 50/70. The volumetric properties of the mixture and some standard properties of the bitumen are given in Table 1.

**Figure 2:**
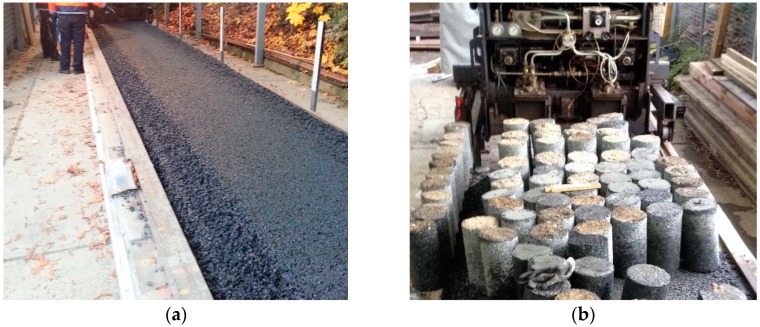
The preparation of the asphalt specimens. (**a**) Test track. (**b**) Cylindrical cores.

The asphalt specimens were obtained from a test track constructed with SMA-11S. The asphalt mixture was prepared by a paver which maintains a paving temperature above 150 °C. The geometry of the test track is 26, 1.2, and 0.3 m in length, width, and depth, respectively, as shown in Figure 2a. Cylindrical cores with 150 mm diameter and 300 mm height were drilled from the test and are illustrated in Figure 2b.

### 2.2. X-ray Computed Tomography Scanning and Digital Image Processing

**Figure 3:**
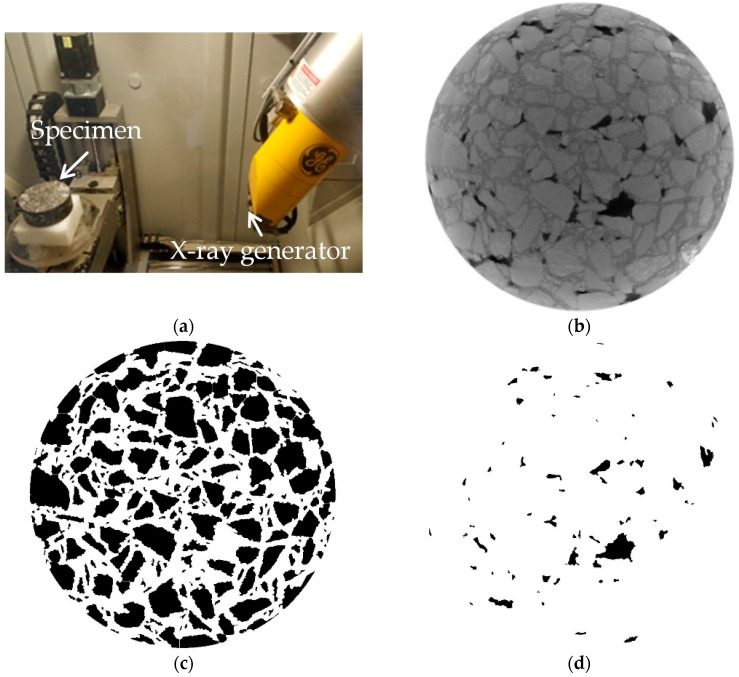
The X-ray CT scanning and digital image processing (DIP). (**a**) X-ray CT scanner. (**b**) Origin gray image. (**c**) Binary image of aggregates. (**d**) Binary image of air voids.

The drilled cores were cut into cylinders with the geometry of 100 mm diameter and 40 mm height. The internal microstructure of the asphalt mixtures was detected through X-ray Computed Tomography (CT) scanning (Phoenix v|tome|x s, GE Sensing and Inspection Technologies GmbH, Wunstorf, Germany); the device is shown in Figure 3a. Scanning intervals were set to 0.1 mm. The resolution of the gray images was 1024 × 1024 pixel^2^ with each pixel being 80 μm. The gray values determined by the X-ray CT device range from 0 to 255 according to material density. Within the asphalt mixtures, aggregate resulted in the maximum gray value while air voids returned the minimum gray values. An origin gray image is shown in Figure 3b. The microstructure was extracted by means of DIP to reconstruct the microstructure for the Finite Element (FE) simulation. The gray images were converted into binary images of aggregates and air voids, as shown in Figure 3c,d, respectively. Details can be found in the previous research [21].

### 2.3. Determination of Non-Uniform Tire Loads

**Figure 4:**
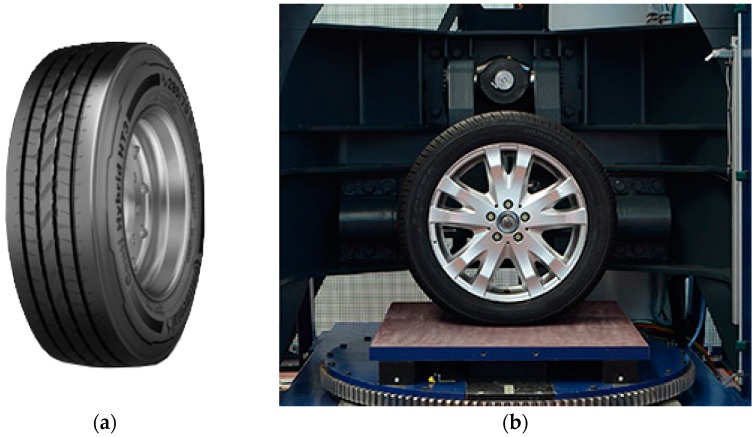
Determination of non-uniform tire load. (**a**) Tire. (**b**) Tire stiffness test rig “SteiReP”.

The non-uniform load is a tire impression with the name Continental ContiHybrid HT3 385-65R22.5 160K, as shown in Figure 4a. For sensing the contact patch dimension and the pressure distribution in the contact patch, the system consists of a static tire stiffness test rig with an additional pressure-sensing pad, as shown in Figure 4b. The test rig is able to measure tires for passenger cars as well as commercial vehicle tires up to a diameter of 1430 mm. The maximum width of mountable tires is about 380 mm. To measure the contact patch dimension and the pressure distribution, the test rig can apply a vertical load up to 40 kN while the inclination angle can be adjusted in a range between ±9°. The measurement itself is a snapshot of the pressure distribution on the pressure sensing pad as the test rig applies a constant wheel load and inclination angle. 

The pressure range of the sensing pad was about 0.7–20.7 bar with an accuracy of ±10% at full load. Furthermore, the spatial resolution of the sensing pad was 1.6 mm, which leads to 65,536 sensing points on an area of 406.4 × 406.4 mm². The resolution was high enough for imaging each separate tread block. With a maximum sample rate of 6.2 frames per second, the sensing pad was able to record the contact patch development for quasi-static measurements. The measuring principle was based on the change of the capacitance in every sensing point when a normal stress was applied.

**Figure 5:**
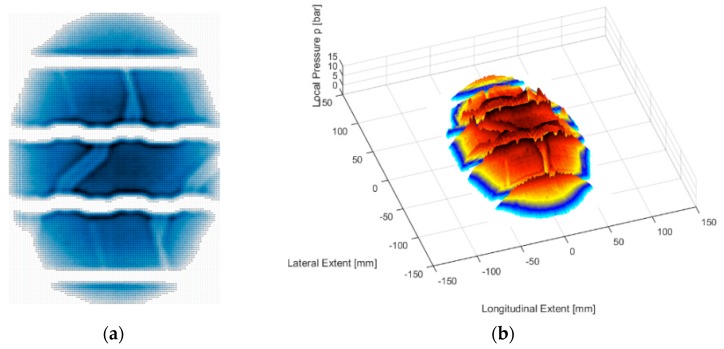
Representation of the contact pressure in (**a**) two-dimension, (**b**) three-dimension.

The evaluation of the measurements was based on a MATLAB (R2015a, MathWorks, Natick, MA, United States) routine to calculate different characteristics. Each sensing point delivers information about the local contact pressure. In respect to the spatial resolution, it is possible to calculate the whole contact area. Other calculable characteristics are the mean contact pressure, the maximum contact pressure, and the length and width of the contact area. A final two-dimensional plot shows the contact patch in lateral and longitudinal extension, as shown in Figure 5a. Furthermore, the contact pressure was represented on the vertical scale in the three-dimensional plot, as shown in Figure 5b.

### 2.4. Numerical Simulation of Uniaxial Compression Test

**Figure 6:**
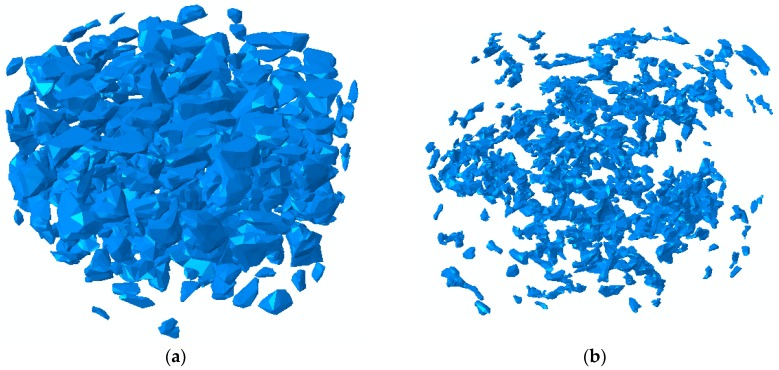
Shell models. (**a**) Aggregates. (**b**) Air voids.

For an explicit representation of aggregate grains in the 3D model, the threshold size to distinguish coarse aggregate from fine aggregate was 2.36 mm, i.e., aggregates with a size smaller than 2.36 mm were ignored in the reconstruction process and were regarded as part of the mortar. It is worth noting that DIP techniques erode some aggregate boundaries in the binary image; thus, the area of aggregate generally shrinks in size, reducing the aggregate content as well [22]. For the construction of the FE model, shell models were first created by stacking the 2D binary images of the aggregates and air voids, as shown in Figure 6. These models only provide the surface morphology of the aggregates and air voids. Therefore, a materialization process had to be done, which assigns solid elements to the shell models. After the materialization, solid models of the two components could be derived and then used in the ABAQUS. The detailed process can be found in previous work [23,24].

The aggregate and asphalt mortar require appropriate material properties for the FE simulation in general-purpose FE software ABAQUS. Compared with the asphalt mortar, aggregates are normally assumed as linear elastic. In this study, a Young’s modulus of 55,000 MPa and a Poisson’s ratio of 0.20 were used [25].

The material properties of asphalt mortar are dependent on temperature, i.e., it is elastic and brittle at low temperature and viscoelastic at higher temperatures. In the range from −5 to 15 °C, the asphalt mortar was considered to be linear viscoelastic by using the generalized Maxwell model for the FE modeling in ABAQUS. The Prony series of asphalt mortar was used for the simulation at −5, 5, and 15 °C.

After importing the geometries of air voids and aggregate grains into ABAQUS, the asphalt mortar was created by means of Boolean operations. The aggregate grains and asphalt mortar were then assembled to construct the microstructural model. Hard contact conditions were assumed for the interaction between aggregates and perfect adhesion was applied in aggregate–mortar interfaces. Because the deformation induced by the loads in this research is believed to be insufficient to cause cracks in the aggregate–mortar interfaces, which are normally considered as the locations of the crack initiation. The asphalt mixture was discretized by linear tetrahedral elements; the element type in ABAQUS was C3D4 and the whole model included 131,642 nodes and 584,680 elements. 

**Figure 7:**
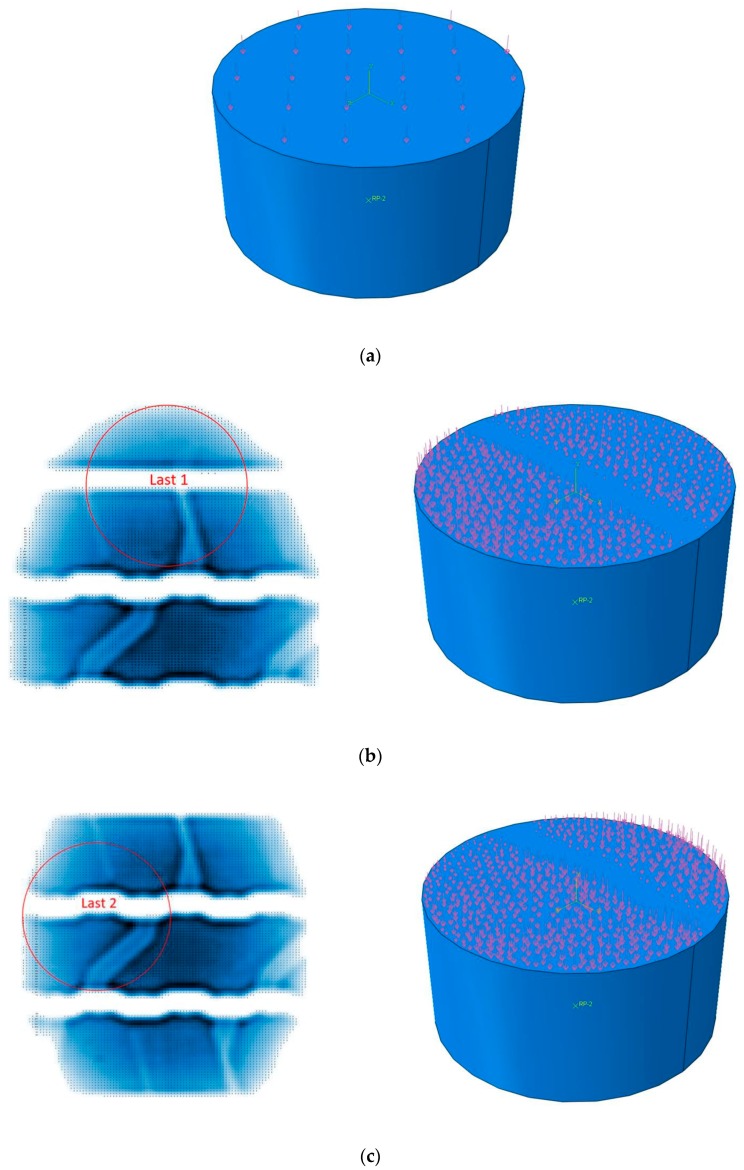
Tire contact patch (**a**) uniform load, (**b**) non-uniform load 1, (**c**) non-uniform load 2.

The modeled cylinder cores were loaded uniaxially with uniform and non-uniform loads on the top face, as shown in Figure 7. Since the cores and the tire footprint did not have the same dimensions, two cutouts from the tire footprint were selected as representative loads to characterize the applications of real loads in the simulation. The uniform comparative loads of the non-uniform load profiles (loads 1 and 2) were found to be 5.3 and 6.5 MPa, respectively. The uniform and non-uniform loads were instantly applied at the beginning of the simulation and then lasted for 5 s.

**Figure 8:**
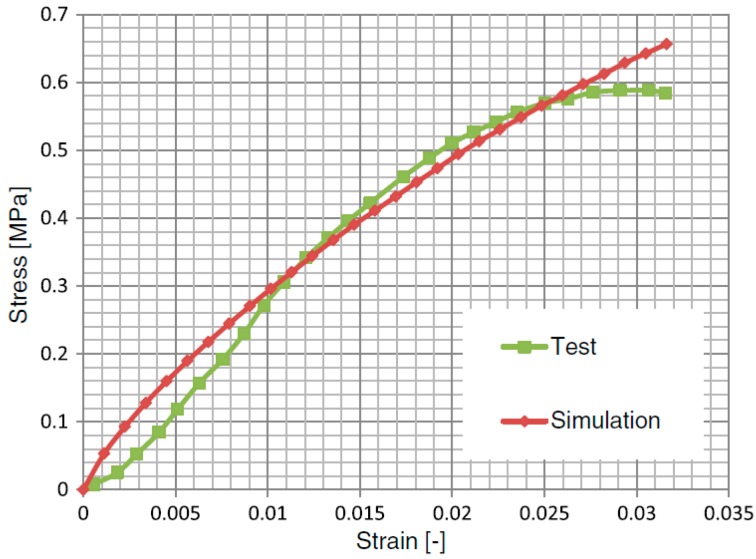
Comparison between experimental and numerical results.

The opposite face was fixed in the longitudinal direction; lateral deformations were permitted. To determine the influence of temperature on the mechanical response of the asphalt mixture, the test was performed at −5, 5, and 15 °C. The reliability of the FE models has been validated in a previous investigation [22]. The computational and experimental stress–strain curves derived from the specimens under uniform load are plotted in Figure 8. The numerical prediction was generally consistent with the experiment within the observed testing period. Moreover, the computational results in this study focused on the variations in the mechanical response due to different loading distributions at different temperatures; thus, the absolute values were less crucial.

## 3. Results and Discussion

### 3.1. Comparison of the Results between Uniform and Non-Uniform Loads

**Figure 9:**
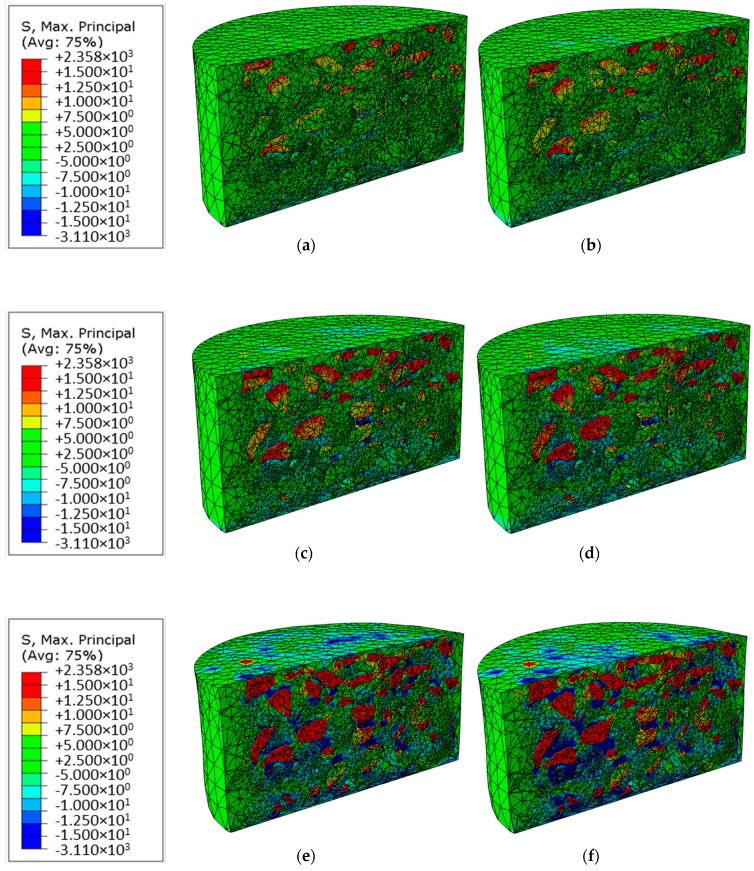
Comparison of the visual representation of the maximum principal stress distribution, (**a**) under uniform load at −5 °C, (**b**) under non-uniform load at −5 °C, (**c**) under uniform load at 5 °C, (**d**) under non-uniform load at 5 °C, (**e**) under uniform load at 15 °C, (**f**) under non-uniform load at 15 °C.

The maximum principal stress theory states that failure in any material, such as asphalt mixtures, occurs when the principal stress in that material due to any loading exceeds the principal stress. Therefore, the maximum principal stress distributions from the uniform and non-uniform (load 2) loads at different temperatures are compared in Figure 9. The results were derived from the end of the simulations, i.e., the moment of 5 s of the simulations.

From Figure 9, one can see a significant increase of the maximum principal stress in both tension and compression directions with the increase of temperature due to decrease of the stiffness of the asphalt mixtures. The concentrations of the maximum principal stresses occur near aggregates, especially around the sharp corners of the aggregates. Particularly, at −5 °C, one can see the relatively large deformation (especially the tensile stress) mainly occurs in the upper two-third region of the asphalt mixtures. With increase of the temperature, the deformation extends to the whole depth of the asphalt mixtures and the compression stress becomes significant.

When comparing the maximum principal stress between the uniform and non-uniform loads, some critical locations with higher maximum principal stresses in the asphalt mixtures under the non-uniform load can be seen. The stress-free areas present in the tire footprint between the tread elements, thereby reducing the loading area and resulting in generally higher deformation at the same loading force. In the model with a temperature of −5 °C, the differences between the different types of the load are relatively small. With increase of the temperature, the differences become more clearly visible. Models with the non-uniform loads show stronger compressive stresses in the asphalt mortar. It is worth noting that the stresses in the asphalt mixtures under the load-free area of the non-uniform loads are still comparable with those in the corresponding area under the uniform loads. This is because of the influence of the adjacent areas with larger deformation under the non-uniform loads.

To quantitatively analyze the difference of the maximum principal stress between the uniform and non-uniform loads, the maximum and average values are compared in Table 2 and Table 3. 

In Table 2, one can see that, at the low temperature of −5 °C, the relative difference between the uniform and non-uniform is largest, and the non-uniform loading model has much higher tensile stress. The tensile stress will cause adhesion failure between aggregates and mortar, which indicates that the non-uniform loading should be paid more attention in the pavement design especially at low temperature.

The differences of the average values are not significant compared to the maximum values, although the absolute average values derived from the models under the non-uniform loads are still larger than those under the uniform loads, as shown in Table 3. Especially the tensile stresses derived from the models under the non-uniform loads may cause more significant adhesion failure, which should be considered in the pavement design algorithm in the future.

### 3.2. Comparison of the Results between the Non-Uniform Loads

The visual differences of the maximum principal stress distributions between the non-uniform loads (loads 1 and 2) are described and interpreted in this section. The results were also derived from the end of the simulations, i.e., the moment of 5 s of the simulations.

**Figure 10:**
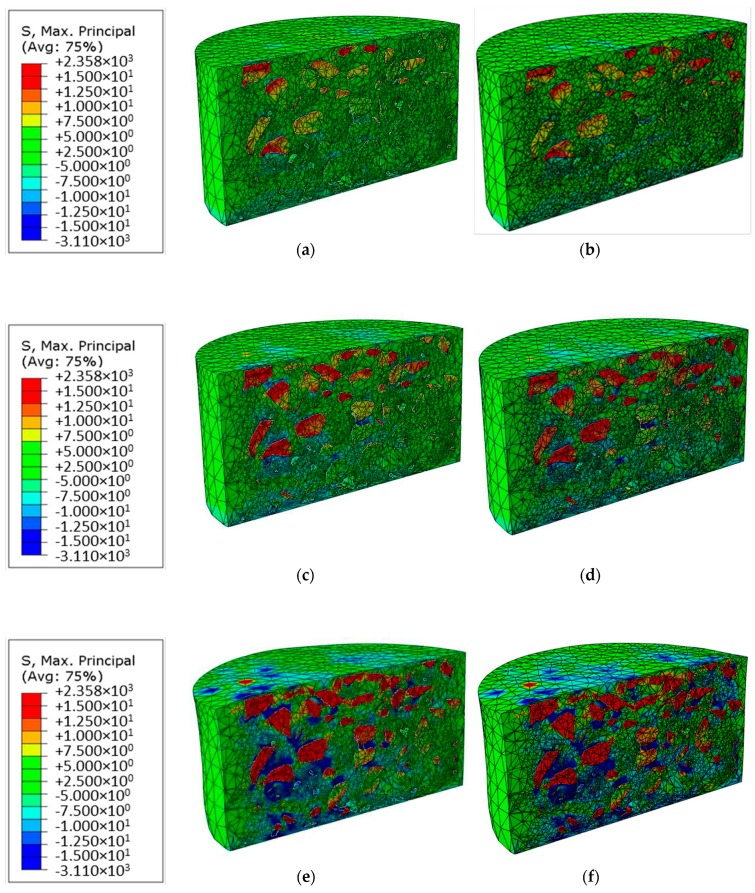
Comparison of the visual representation of the maximum principal stress distribution, (**a**) under load 1 at −5 °C, (**b**) under load 2 at −5 °C, (**c**) under load 1 at 5 °C, (**d**) under load 2 at 5 °C, (**e**) under load 1 at 15 °C, (**f**) under load 2 at 15 °C.

Some similar conclusions mentioned in the last section can also be derived from Figure 10. The maximum principal stress in both tension and compression directions increases with the increase of temperature. The concentrations of the maximum principal stresses occur around the sharp corners of the aggregates. The deformation extends to the whole asphalt mixtures and the compression stress becomes significant with increase of the temperature.

Moreover, for the two models at each temperature, in the left region of both the models, relatively strong tensile stresses can be seen around the aggregates. In the right region of both the models, the tensile stresses in the model under load 1 were significantly less than those under load 2. This is because the average loading pressure of load 1 (5.3 MPa) was smaller than that of load 2 (6.5 MPa). 

In summary, between two non-uniform loads, the differences of the mechanical responses should not be neglected. Even at a very low temperature, such as at −5 °C, one can already see significant differences between the stress distributions of the models. These are reinforced by increasing the temperature. Particularly in the region of the right part of the models, strong differences between the models can be seen. That means even the non-uniform loads are considered in the pavement design algorithm, the difference between the non-uniform loads is still large and should be taken into account.

The difference of the maximum principal stress between the non-uniform loads is further quantitatively analyzed, and the maximum and average values are compared in Table 4 and Table 5, respectively. 

Similar conclusions can be found. At the low temperature (−5 °C), the relative difference of the maximum and average stresses between the non-uniform loads is largest. The most differences between the tensile stresses derived from the non-uniform loads are larger than those from the uniform load and non-uniform load 2, which indicates that the different type of the non-uniform loading should be paid attention in the pavement design.

## 4. Conclusion and Outlook

In this study, a 3D finite element model was reconstructed from X-ray CT images. The model was loaded with uniform and non-uniform load distributions at three different temperatures. The results are summarized below.

For both models loaded with uniform and non-uniform loads, a significant increase of the maximum principal stress was seen in both tension and compression directions with the increase of temperature. When comparing the maximum principal stresses of the uniform and non-uniform loads, some critical locations with higher maximum principal stresses in the asphalt mixtures under the non-uniform load can be seen. With increase of the temperature, the differences become more clearly visible. The non-uniform loading model has much higher tensile stress. The tensile stress will cause adhesion failure between aggregates and mortar, which indicates that the non-uniform loading should be paid more attention in the pavement design especially at low temperature.

Comparing the models loaded with different non-uniform loads, the differences should not be neglected. That means even the non-uniform loads are considered in the pavement design algorithm, the different distribution between the non-uniform loads is still large and should be taken into account.

These conclusions form the basis for further investigations. A follow-up of this approach presented here may offer a significant contribution to make the design method more realistic by means of appropriate simulations and to enable more reliable prediction. Studies are to be carried out, wherein the following should be considered. All components of the tire−pavement interaction should be considered. A statistical analysis regarding the different microstructural FE models and the pressure distributions should be performed. Furthermore, advanced FE models, such as consideration of fracture behavior using cohesive zone method (CZM), should be developed [26].

## Figures and Tables

**Table 1:** Data about the mix design and bitumen.

Volumetric Values of the Mix Design		Penetration and Viscosity Measurements of Bitumen	
Parameter	Value	Parameter	Value
Bitumen content (wt.%)	6.9	Penetration at 25 °C (1/10 mm)	52
Bulk density of Marshall specimens (kg/m^3^)	2.415	Ring and Ball (softening point (°C)	51.4
		Fraass breaking point (°C)	Max. −8
Void ratio of Marshall specimens (%)	2.9341	Dynamic viscosity at 60 °C (Pa.s)	Min. 145
		Kinematic viscosity at 135 °C (Pa.s)	Min. 295

**Table 2:** Comparison of maximum stresses as a function of temperature and type of load.

**Compression Stress**	**T = −5 °C**	**T = 5 °C**	**T = 15 °C**
Uniform (MPa)	−41.95	−143.32	−437.13
Non-uniform (MPa)	−71.38	−188.75	−629.55
Absolute difference (MPa)	−29.43	−45.43	−192.42
Relative difference (%)	70.15	31.70	44.02
**Tensile Stress**	**T = −5 °C**	**T = 5 °C**	**T = 15 °C**
Uniform (MPa)	325.82	362.27	1755.33
Non-uniform (MPa)	575.14	586.04	1908.70
Absolute difference (MPa)	249.32	223.77	153.37
Relative difference (%)	76.52	61.77	8.74

**Table 3:** Comparison of average stresses as a function of temperature and type of load.

**Compression Stress**	**T = −5 °C**	**T = 5 °C**	**T = 15 °C**
Uniform (MPa)	−2.18	−5.08	−14.33
Non-uniform (MPa)	−2.36	−5.52	−16.09
Absolute difference (MPa)	−0.18	−0.44	−1.76
Relative difference (%)	8.26	8.66	12.28
**Tensile Stress**	**T = −5 °C**	**T = 5 °C**	**T = 15 °C**
Uniform (MPa)	2.17	4.30	12.40
Non-uniform (MPa)	2.52	4.85	14.07
Absolute difference (MPa)	0.35	0.55	1.67
Relative difference (%)	16.13	12.79	13.47

**Table 4:** Comparison of maximum stresses as a function of temperature and type of load.

**Compression Stress**	**T = −5 °C**	**T = 5 °C**	**T = 15 °C**
Load 1 (MPa)	−49.18	−138.81	−548.16
Load 2 (MPa)	−71.38	−188.75	−629.55
Absolute difference (MPa)	−22.2	−49.94	−81.39
Relative difference (%)	45.14	35.98	14.85
**Tensile Stress**	**T = −5 °C**	**T = 5 °C**	**T = 15 °C**
Load 1 (MPa)	160.67	434.71	1400.69
Load 2 (MPa)	275.14	586.04	1908.70
Absolute difference (MPa)	114.47	151.33	508.01
Relative difference (%)	71.25	34.81	36.27

**Table 5:** Comparison of average stresses as a function of temperature and type of load.

**Compression Stress**	**T = −5 °C**	**T = 5 °C**	**T = 15 °C**
Load 1 (MPa)	−1.88	−4.39	−12.76
Load 2 (MPa)	−2.36	−5.52	−16.09
Absolute difference (MPa)	−0.48	−1.13	−3.33
Relative difference (%)	25.53	25.75	26.10
**Tensile Stress**	**T = −5 °C**	**T = 5 °C**	**T = 15 °C**
Load 1 (MPa)	1.94	3.77	11.08
Load 2 (MPa)	2.52	4.85	14.07
Absolute difference (MPa)	0.58	1.08	2.99
Relative difference (%)	29.90	28.65	26.99
